# Prevalence of dyslipidemia among HIV-infected patients using first-line highly active antiretroviral therapy in Southern Ethiopia: a cross-sectional comparative group study

**DOI:** 10.1186/1742-6405-9-31

**Published:** 2012-10-25

**Authors:** Agete Tadewos, Zelalem Addis, Henock Ambachew, Sandip Banerjee

**Affiliations:** 1 Referral hospital laboratory, Hawassa University College of Medicine and Health Science, Hawassa, Southern Ethiopia; 2 School of Biomedical and Laboratory Sciences, University of Gondar College of Medicine and Health Science, P.O Box 196, Gondar, Ethiopia; 3 Department of Medical laboratory Technology, Hawassa University College of Medicine and Health Science, Hawassa, Southern Ethiopia; 4 Department of Animal and Range, Hawassa University College of Agriculture Sciences, Hawassa, Southern Ethiopia

**Keywords:** Dyslipidemia, HIV/AIDS, Antiretroviral therapy, *Ethiopia*

## Abstract

**Background:**

Data on lipid profile abnormalities among patients receiving highly active antiretroviral treatment in Ethiopia are very limited. The aim of this study was to determine the prevalence of dyslipidemia and characteristics of lipid profiles among patients living with human immunodeficiency virus (HIV) using first-line highly active antiretroviral therapy (HAART) in Southern Ethiopia.

**Methods:**

This cross sectional comparative group study was conducted between March and May 2012, and included 113 HIV infected patients treated for a minimum of one year with first-line HAART regimens that included Efavirenz and Nevirapine (HAART group) and others 113 who had never received HAART (pre-HAART group). Serum lipid profiles were determined after overnight fasting and dyslipidemia was assessed according to the United State National Cholesterol Education program-III guideline. For statistical analysis Chi-square, student’s t-test, and logistic regression were used using Statistical Package for Social Sciences (SPSS) Version 20.

**Result:**

Ninety-three (82.3%) of HAART and 87 (76.9%) pre-HAART patients had at least one laboratory abnormality, which is compatible with a diagnosis of dyslipidemia. Total cholesterol ≥ 200 mg/dl occurred in 43.4% of HAART and 15.9% pre-HAART patients (p=<0.0001), whereas HDL-cholesterol below 40 mg/dl occurred in 43.4% and in 63.7% respectively, (p=0.002). The LDL-cholesterol ≥ 130 mg/dl occurred in 33.6% of HAART and 15% pre-HAART patients (p=0.001), while triglycerides ≥ 150 mg/dl occurred in 55.8% and 31.0% respectively, (p=0.001). Receiving of HAART was significantly and positively associated with raised total cholesterol, LDL-cholesterol, and triglycerides. The adjusted odds ratio (95% CI) of HAART-treated vs. pre-HAART was 3.80 (1.34-6.55) for total cholesterol ≥ 200 mg/dl; 2.64 (1.31-5.32) for LDL- cholesterol ≥ 130 mg/dl and 2.50 (1.41-4.42) for triglycerides ≥150 mg/dl.

**Conclusion:**

Use of first-line antiretroviral therapy regimens that contain Efavirenz and Nevirapine were associated with raised total cholesterol, LDL-cholesterol, and triglycerides, an established atherogenic lipid profiles. Lipid profiles should be performed at baseline before commencement of antiretroviral therapy and then periodically through treatment follow-up to monitor any rising trends.

## Introduction

In 2011, an estimated 34 million people were living with human immunodeficiency virus /acquired immunodeficiency syndrome (HIV/AIDS) worldwide; of them 22.9 million were living in Sub-Saharan Africa. About 1.2 million people were estimated to be living with HIV in Ethiopia [1].The introduction of highly active antiretroviral therapy (HAART) has led to a marked reduction in AIDS-related morbidity and mortality [2]. Since its introduction patients have started to live longer, however co-morbid problems have been emerged. Dyslipidemia, insulin resistance, and diabetes are some of metabolic complications of long-term use of HAART [3]. The characteristics of dyslipidemia in HIV-infected patients receiving HAART includes, elevated level of total cholesterol (TC), LDL-cholesterol (LDL-c), triglycerides (TG), and decreased HDL-cholesterol (HDL-c), include with severe hypertriglyceridemia in some patients [4]. Some antiretroviral drugs, such as stavudine (d4T) [5], and protease inhibitors (PIs) [6], increase the blood levels of TC, LDL-c, and TGs with variable effects on levels of HDL-c. Nevirapine (NVP) use is associated with increases in LDL-c [7], whereas increases in TC and TG are observed with use of efavirenz (EFV), particularly with longer duration of therapy [8]. Therefore, the use of HAART raises concerning metabolic disorders and cardiovascular risk in HIV infected patients who now present an extended life expectancy [9]. The prevalence of dyslipidemia in resource-limited settings has not been well characterized and current World Health Organization (WHO) antiretroviral therapy (ART) guidelines do not include a recommendation that lipid monitoring should be conducted in patients receiving first-line HAART [10]. In addition, evidences in support of dyslipidemia associated with first-line HAART that include EFV and NVP in Sub-Sahara African countries are scarce [11,12].The aim of the present study was to determine the prevalence of dyslipidemia and characteristics of lipid profiles among people living with HIV infection receiving first-line HAART in Southern Ethiopia.

## Methods

### Study setting and study population

This was a cross-sectional comparative group study. Subjects were recruited between March 2012 and May 2012 at ART clinic of Hawassa University Referral Hospital. Two groups of participants were selected for this study. The first group included HIV-infected individuals who had been receiving WHO recommended first-line HAART for a minimum of one year (HAART group). Participants used first-line HAART regimens that included nucleoside reverse transcriptase inhibitors (NRTIs): lamivudine (3TC), ZDV, or d4T, and non-nucleoside reverse transcriptase inhibitors (NNRTIs): NVP or EFV. Patients who had had their therapy regimens changed during follow-up were not included. The second group was pre-tested and confirmed HIV-positive individuals who were not yet getting HAART. All participants included were ≥18 years of age and HAART treated group to have a good ART adherence (adherence rate ≥ 95%). A good adherence is defined by missing < 2 dose of 30 doses or < 3 dose of 60 doses; and it was adopted from Ethiopian Federal Ministry of Health, HIV Care/ART follow-up form. Participants receiving lipid altering therapies, pregnant women, known diabetes mellitus patients and renal failures were excluded.

### Assessments and measurements

For all participants, data were collected on the socio-demographic informations along with body mass index, medical history including diabetes mellitus, renal failures, use of drugs that alter lipid profiles, and current use of anti-TB drugs. CD4^+^ lymphocyte count was done by using flow cytometry instrument (Becton Dickinson, CA, USA). Blood sample was collected from each participant after a 12 hour overnight fast and centrifuged at 3000 cycles/ minute, and then serum was obtained for lipid profiles. Fasting serum TC and TG were assayed by enzymatic method (Linear chemicals, Montgat, Spain). Serum HDL-c determined by enzymatic method after a selective precipitation of apolipoprotein containing lipoproteins (very low density lipoprotein, LDL-c, and apolipoprotein a (Lpa) by phosphotungstic acid/MgCl_2_ (Linear chemicals, Montgat, Spain). LDL-c was assessed by enzymatic method after precipitation of LDL by polyvinyl sulfate (Linear chemicals, Montgat, Spain) and then LDL-c was calculated by subtracting the supernatant cholesterol fractions from the TC of the sample. And also the TC/HDL-c ratio was calculated.

Finally dyslipidemia was defined as TC ≥ 200 mg/dl, HDL-c < 40 mg/dl, LDL-c ≥ 130 mg/dl, TG ≥ 150 mg/dl and TC/HDL-c ratio ≥ 5 by the United States National Cholesterol Education Program, Adult Treatment Panel (NCEP-ATP) III guidelines [13].

### Statistical analysis

The estimation of the sample size was based on the difference between proportions and the following parameters were considered: alpha (α) = 5%, beta (β) = 10 % and power = 90%. Concerning dyslipidemia**,** we considered frequencies of LDL-c ≥130 mg/dl, which was 21% for the group of individuals with HIV/AIDS without HAART treatment [14]; and 40.8% for patients receiving HAART treatment [15]. With these parameters, the sample size calculated was 230 participants (115 pre-HAART and 115 HAART-treated patients).Data entry and Database management was completed using EPI-INFO 2002. Statistical analyses were done using Statistical Package for Social Sciences (SPSS) Version 20. Chi-square test was used to evaluate differences in frequency distribution. Student’s t-test was applied to assess differences between two means. Logistic regression was also used to determine the association of independent factors (age, gender, and treatment status, type of treatment, current CD4 counts, and history of smoking, body mass index and current use of anti-TB drugs) with abnormal level of each lipid profile. P-value less than 0.05 considered as statistically significant at 95% confidence interval (CI).

### Ethical consideration

The study was approved by the ethical review committee of School of Biomedical and Laboratory Sciences, University of Gondar. All individuals participated in the study provided their informed consent.

## Results

### General characteristics of study participants

Total number of participants enrolled in the study was 226, with 79 (35%) males and 147 (65%) females. Two groups were studied: the first group 113 HIV-infected patients (38 (33.6%) males, 75 (66.4%) females) were currently receiving HAART treatment; the second group 113 patients (41 (36.3%) males, 72 (63.7%) females) were pre-HAART. The age of individuals who were on HAART was 37.2 ± 8.7 while that of pre-HAART was 33.7 ±8.3 years and the age difference was significantly different (Table 1). First-line HAART regimens were a combinations of 2NRTI and 1NNRTI. All regimens included 3TC. HAART patients had been on treatment for a mean of 49.4 months (standard deviation: 16.5). The number of patients on ZDV/3TC/EFV, and ZDV/3TC/NVP regimens were 22 (19.5%) and 36 (31.8%) respectively, while those on d4T/3TC/EFV, and d4T/3TC/NVP regimens were 24 (21.2%) and 31 (27.4%) respectively. Accordingly, 58 (51.3%) patients were on ZDV, 55 (48.7%) were on d4T, 46 (40.7%) were on EFV, and 67 (59.3%) were on NVP.

**Table 1:** Characteristics of study population by HAART status in Hawassa, Southern Ethiopia

**Variables**	**HAART group (n=113)**	**pre-HAART group (n=113)**
Gender = Female, n (%)	75 (66.4)	72 (63.7)
Male, n (%)	38 (33.6)	41 (36.3)
Age, (years), mean ± SD	37.2 ( 8.7)	33.7 (8.3)**
18–30 years, n (%)	26 (23)	49 (43.4)
31–40 years, n (%)	59 (52.2)	47(41.6)
41–50 years, n (%)	21 (18.6)	12 (10.6)
>50 years, n (%)	7 (6.2)	5 (4.4)
BMI (Kg/m^2^), mean ± SD	22.3 (4.1)	21.5 ( 3.9)
<18 Kg/m^2^, n (%)	8 (7)	14 (12.4)
18–25 Kg/m^2^, n (%)	82 (72.6)	84 (74.3)
>25 Kg/m^2^, n (%)	23 (20.4)	15 (13.3)
CD4+ count (cells/mm^3^), mean ± SD	460 (221)	356 (216.7) ***
< 200 cells/mm^3^, n (%)	16 (14.2)	29 (25.7)
200–400 cells/mm^3^, n (%)	32 (28.3)	44 (38.9)
> 400 cells/mm^3^, n (%)	65 (57.5)	40 (35.4)
Duration of HAART, mean ±SD	49.4 (16.5)	--
12–24 months, n (%)	10 (8.8)	--
24–36 months, n (%)	18 (15.9)	--
36–48 months, n (%)	27 (23.9)	--
48–60 months, n (%)	24 (21.2)	--
> 60 years, n (%)	34 (30.1)	--
Current anti-TB users, n (%)	0	4 (3.5)
Current smoking, n (%)	6 (5.3)	7 (6.2)
Past smoking, n (%)	15 (13.3)	13 (11.5)

BMI, body mass index; HAART, highly active antiretroviral therapy; mo, month; TB, tuberculosis; SD, standard deviation; ** p-value < 0.01; *** p-value < 0.001.

### Dyslipidemia and characteristics of lipid profiles

**Figure 1:**
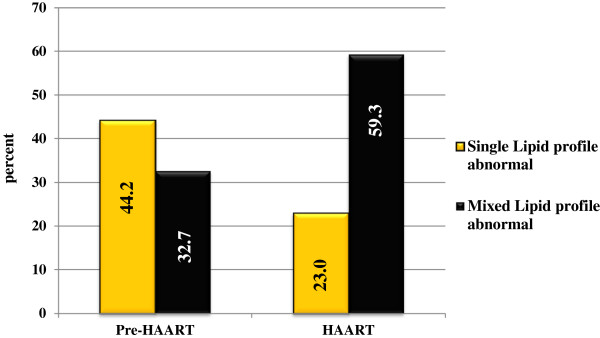
**Category of dyslipidemia among study population by HAART status in Hawassa, Southern Ethiopia.** Number of patients in HAART group was 113 and in pre-HAART group were113; number of patients with single lipid profile abnormal in pre-HAART group and HAART group were respectively, 50 and 26; and number of patients with mixed lipid profiles abnormal in pre-HAART group and HAART group were respectively, 37 and 67; HAART, highly active antiretroviral therapy; (single lipid profile abnormal means only one lipid parameter is abnormal and mixed lipid profile abnormal means more than one lipid parameters abnormal within a single individual).

Ninety-three (82.3%) of HAART treated and 87 (76.9%) pre-HAART patients had at least one laboratory abnormality, which is compatible with a diagnosis of dyslipidemia (Figure 1). The prevalence of TC ≥ 200 mg/dl, LDL-c ≥130 mg/dl and TG ≥150 mg/dl were significantly higher in HAART group when compared to pre-HAART group. The prevalence of HDL-c below 40 mg/dl was significantly higher in pre-HAART group when compared to those on HAART. However, there was no significant difference in TC/HDL-c ratio between HAART treated and pre-HAART patients (Table 2).

**Table 2:** Serum lipid profiles of study population by HAART status in Hawassa, Southern Ethiopia

**Parameters**	**HAART group (n=113)**	**Pre-HAART group (n=113)**
Total cholesterol, mean ± SD	199.6 (51.5)	155.4 (44) ***
< 200mg/dl	64 (56.6%)	95 (84.1%)
≥ 200mg/dl	49 (43.4%)	18 (15.9%) ***
HDL-cholesterol, mean ± SD	43.2 (14.3)	33.3 (12.8 ) ***
< 40mg/dl	49 (43.4%)	72 (63.7%) **
≥ 40mg/dl	64 (56.6%)	41 (36.3%)
LDL-cholesterol, mean ± SD	119.3 (43)	97.3 (36.2) ***
< 130mg/dl	75 (66.4%)	96 (85%)
≥ 130mg/dl	38 (33.6%)	17 (15.0%) **
Triglycerides, mean ± SD	204 (143)	138 (76 ) ***
< 150mg/dl	50 (44.2%)	78 (69%)
≥150mg/dl	63 (55.8%)	35 (31.0%) ***
TC/HDL-c ratio, mean ± SD	5.2 (2.4)	5.3 (2.6)
< 5	62 (54.9%)	64 (56.6%)
≥ 5	51 (45.1%)	49 (43.4%)

HAART, highly active antiretroviral therapy; TC, total cholesterol; HDL, high-density lipoprotein; LDL, low- density lipoprotein; SD, standard deviation;** p-value < 0.01; *** p-value <0.001.

### Dyslipidemia and first-line HAART

**Figure 2:**
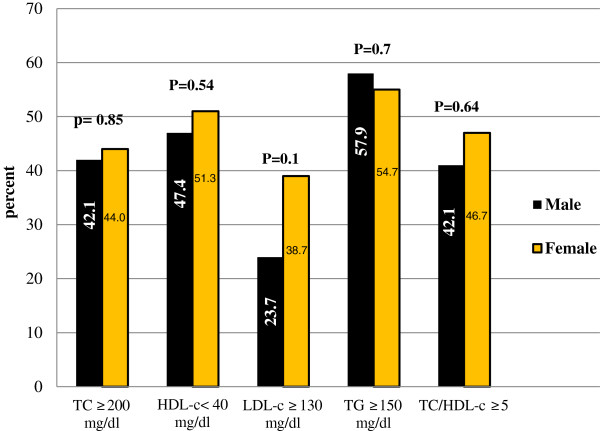
**Gender and abnormal lipid profiles among first-line HAART treated patients in Hawassa, Southern Ethiopia.** [Males (n=38); females (n=75); TC, total cholesterol; HDL-c, high-density lipoprotein cholesterol; LDL-c, low-density lipoprotein cholesterol; TG, triglyceride].

No significant difference observed in lipid profile derangements between patients receiving ZDV when compared to those on d4T; and patients treated with EFV when compared to those treated with NVP (Table 3 and Table 4). In addition, no significant difference observed in lipid derangements between HAART treated males and females; however, the proportion of raised LDL-c is slightly higher in females when compared to males (Figure 2).

**Table 3:** Prevalence of abnormal lipid profiles among patients treated with NRTIs based first-line antiretrovirals in Hawassa, Southern Ethiopia

**Lipid profile**	**ZDV-based n=58 (%)**	**d4T-based n=55 (%)**	***p-value***
TC ≥ 200 mg/dl	25 (43.1)	24 (43.6)	0.95
HDL-c < 40 mg/dl	24 (41.4)	25 (45.4)	0.66
LDL-c ≥ 130 mg/dl	20 (34.5)	18 (32.7)	0.84
TG ≥ 150 mg/dl	32 (55.2)	31 (56.4)	0.41
TC/HDL-c ratio ≥5	24 (41.4)	27 (49.1)	0.89

TC, total cholesterol; HDL-c, high-density lipoprotein cholesterol; LDL-c, low-density lipoprotein cholesterol; TG, triglyceride; ZDV, Zidovudine; d4T, stavudine; p, significance level.

**Table 4:** Prevalence of abnormal lipid profiles among patients treated with NNRTIs based first-line antiretrovirals in Hawassa, Southern Ethiopia

**Lipid profile**	**EFV-based n=46 (%)**	**NVP-based n=67 (%)**	**P value**
TC > 200 mg/dl	18 (39.1)	31 (46.3)	0.45
HDL-c < 40 mg/dl	22 (47.8)	27 (40.3)	0.43
LDL-c > 130 mg/dl	14 (30.4)	24 (35.8)	0.55
TG > 150 mg/dl	25 (54.3)	38 (56.7)	0.80
TC/HDL ratio >5	22 (47.8)	29 (43.3)	0.63

TC, total cholesterol; HDL-c, high-density lipoprotein cholesterol; LDL-c, low-density lipoprotein cholesterol; TG, triglyceride; EFV, Efavirenz; NVP, Nevirapine; p, significance level.

### Dyslipidemia and risk factors

Multivariate analysis was adjusted for potential confounding factors such as age, sex, current history of smoking, past history of smoking, BMI, CD4+cells, current use of anti-TB drugs, and HAART treatment. Receiving of HAART was significantly and positively associated with raised TC, LDL-c, and TG. The adjusted odds ratio (95% CI) of HAART-treated vs. pre-HAART was 3.80 (1.34-6.55) for TC ≥200 mg/dl; 2.64 (1.31-5.32) for LDL-c ≥ 130 mg/dl and 2.50 (1.41-4.42) for TG ≥ 150 mg/dl. However, antiretroviral therapy was not positively associated with decreased of HDL-cholesterol (Table 5).

**Table 5:** Associations HAART and other variables with dyslipidemia among HIV-infected patients in Hawassa, Southern Ethiopia

**Explanatory variable**	**Outcome**	**variables**		
	**TC ≥ 200 mg/dl**	**HDL< 40 mg/dl**	**LDL ≥ 130 mg/dl**	**TG ≥ 150 mg/dl**
	**AOR (95% CI)**	**AOR (95% CI)**	**AOR (95% CI)**	**AOR (95% CI)**
CD4^+^cells ≥200/mm^3^*	1.00	1.00	1.00	1.00
CD4^+^cells < 200/mm^3^	0.91 (0.39-2.10)	1.95 (0.94-4.07)	0.89 (0.37-2.15)	0.87 (0.42-1.80)
P-value	0.82	0.07	0.79	0.71
BMI ≤ 25 Kg/m^2^*	1.00	1.00	1.00	1.00
BMI > 25 Kg/m^2^	2.96 (1.35-6.49)	0.55 (0.26-1.18)	2.66 (1.17-5.59)	2.82 (1.31-6.07)
P-value	**0.007**	0.13	**0.02**	**0.008**
Gender = Female*	1.00	1.00	1.00	1.00
Male	0.71 (0.32-1.57)	0.73 (0.38-1.42)	0.53 (0.22-1.25)	1.44 (0.74-2.82)
P-value	0.4	0.36	0.15	0.28
Age ≤ 40 years*	1.00	1.00	1.00	1.00
Age > 40 years	3.04 (1.41-6.55)	2.64 (1.23-5.69)	3.28 (1.48-7.30)	1.47 (0.71-3.05)
P-value	**0.004**	**0.01**	**0.004**	0.29
Pre-HAART*	1.00	1.00	1.00	1.00
HAART	3.80 (1.34-7.44)	0.44 (0.25-0.78)	2.64 (1.31-5.32)	2.50 (1.41-4.42)
P-value	**<0.0001**	**0.005**	**0.006**	**0.002**
No smoking*	1.00	1.00	1.00	1.00
Current smoking	0.90 (0.20-4.00)	2.13 (0.57-8.01)	0.32 (0.36-2.82)	0.75 (0.21-2.64)
P-value	0.89	0.26	0.3	0.65
No smoking*	1.00	1.00	1.00	1.00
Past smoking	0.77 (0.25-2.33)	1.46 (0.57-3.75)	0.56 (0.16-1.91)	0.67 (0.26-1.75)
P-value	0.64	0.43	0.35	0.42
No ant-TB drug *	1.00	1.00	1.00	1.00
Ant-TB drug	1.85(0.17-20.51)	NA	1.74(0.1519.82)	NA
P-value	0.62	NA	0.65	NA

HAART, highly active antiretroviral therapy; AOR, adjusted odds ratio; CI, confidence interval; BMI, body mass index; TC, total cholesterol; HDL, high-density lipoprotein cholesterol; LDL, low-density lipoprotein cholesterol; TG, triglyceride; NA, not available; p, significance level; TB, tuberculosis; * Reference category.

Univariate and multivariate analysis were also applied to assess independent risk factors for each abnormal lipid profile in the subgroup of 113 HAART treated patients. In these both models, age was the risk factor of raised TC and decreased HDL-c; and BMI was the risk factor of raised TC in HAART treated patients (Table 6).

**Table 6:** Associations of variables with abnormal lipid profiles among first-line HAART treated patients in Hawassa, Southern Ethiopia

**Explanatory variables**	**Outcome**	**variables**		
	**TC ≥ 200 mg/dl**	**HDL-c < 40 mg/dl**	**LDL-c ≥ 130mg/dl**	**TG ≥150 mg/dl**
BMI > 25kg/m^2^				
UOR (95% CI)	3.09 (1.18-8.05)*	0.50 (0.19-1.33)	2.68 (1.05-6.85)*	2.71 (0.98-7.50)
AOR (95% CI)	3.21 (1.16-8.91)*	0.49 (0.17-1.39)	2.44 (0.91-6.51)	2.92 (0.99-8.44)
Gender (male)				
UOR (95% CI)	0.93 (0.42-2.04)	1.28 (0.58-2.80)	0.49 (0.20-1.19)	1.14 (0.52-2.51)
AOR (95% CI)	0.68 (0.23-2.04)	0.69 (0.24-1.96)	0.43 (0.13-1.46)	1.09 (0.40-2.99)
Age > 40 years				
UOR (95% CI)	3.13 (1.28-7.63)*	3.14 (1.29-7.64)*	2.08 (0.86-5.00)	1.97 (0.80-4.84)
AOR (95% CI)	4.43 (1.54-12.72)**	4.22 (1.46-12.16)**	4.28 (1.41-12.97)*	2.12 (0.77-5.82)
HAART ≤ 2 years				
UOR (95% CI)	0.95 (0.24-3.75)	0.35 (0.08-1.49)	1.85 (0.37-9.39)	1.00 (0.26-3.97)
AOR (95% CI)	0.76 (0.16-3.66)	0.30 (0.06-1.47)	1.35 (0.22-8.20)	0.92 (0.21-4.09)
Current smoking				
UOR (95% CI)	0.64 (0.11-3.64)	1.33 (0.26-6.87)	0.38 (0.04-3.36)	0.78 (0.15-4.06)
AOR (95% CI)	0.77 (0.12-5.79)	0.70 (0.10-4.80)	0.51 (0.04-5.82)	0.78 (0.12-5.04)
Past smoking				
UOR (95% CI)	1.17 (0.39-3.47)	1.17 (0.39-3.47)	0.45 (0.12-1.70)	1.22 (0.40-3.70)
AOR (95% CI)	0.82 (0.20-3.34)	0.62 (0.16-2.45)	0.39 (0.08-1.98)	0.88 (0.23-3.40)
d4T				
UOR (95% CI)	1.02 (0.48-2.15)	1.18 (0.56-2.49)	0.92 (0.42-2.02	1.05 (0.50-2.21)
AOR (95% CI)	1.29 (0.57-2.93)	1.41 (0.63-3.16)	1.22 (0.52-2.85)	1.17 (0.53-2.58)
EFV				
UOR(95% CI)	0.75 (0.35-1.60)	1.36 (0.64-2.89)	0.78 (0.35-1.75)	0.91 (0.43-1.93)
AOR (95% CI)	0.77 (0.33-1.79)	1.36 (0.60 3.12)	0.91 (0.37-2.22)	0.97 (0.43-2.18)

HAART, highly active antiretroviral therapy therapy; UOR, un adjusted odds ratio; AOR, adjusted odds ratio; CI, confidence interval; TC, total cholesterol; HDL-c, high-density lipoprotein cholesterol; LDL-c, low- density lipoprotein cholesterol; TG, triglyceride; d4T Stavudine; BMI, body mass index; EFV, Efavirenz; * p-value < 0.05; ** p-value < 0.01. **Reference category:** BMI ≤ 25 Kg/m^2^, Females, Age ≤ 40 years, no smoking, Nevirapine group, Zidovudine group; HAART treatment >2years.

## Discussion

The aim of this cross-sectional study carried out in a resource limited East African setting was to assess the prevalence of dyslipidemia (lipid profile derangements) among HIV-infected patients receiving first-line HAART that include NNRTIs. We found that CD4 cell count in the HAART group was significantly different from the pre-HAART group, thus showing the effects of HAART in improving the immunological properties of the subjects.

In the present study, the majority of HAART patients (82.3%) had at least one laboratory abnormality, which is compatible with a diagnosis of dyslipidemia according to NCEP-ATP III criteria. We found that the proportions of raised TC, LDL-c and TG were significantly higher in HAART group when compared to pre-HAART group. The proportion of HDL-c below 40 mg/dl was significantly higher in the pre-HAART group when compared to those on HAART, thus indicating the effects of HAART treatment in raising the lipid profiles, in addition to the restoration of health. However, there was no significant difference between treated group and non-treated group regarding TC/HDL-c ratio, this because increases in TC were counterbalanced by HDL-c improvement. The described raised lipid profiles (TC, LDL-c and TG), are atherogenic [13,16,17], and suggest a potential risk for the development of cardiovascular diseases in a significant proportion of HIV-infected patients in the near future.

The association between lipid derangements and HAART has been mainly depicted for regimens that contain PIs [6,18]. In addition, NNRTIs derange lipid profiles during therapy [7,8]. However, supportive evidences are very scarce in Sub-Sahara African countries concerning lipid derangements in patients receiving NNRTs including regimens [11,12]. We found that the prevalence of raised TC in HAART group was (43.4%). This prevalence rate is higher than the study reported from a resource poor West African setting [19]. The prevalence rate in this study was 4.5%. However, in this study, the cutoff limit was >210 mg/dl; and this value is higher than that of NCEP-ATP III guidelines, as used in our study. Based on HDL-c cut off value, the prevalence of HDL-c in our treatment group was 43.4%. This is comparable with the prevalence rate reported from Spain [20]. However, it is higher than the prevalence reported from rural South India [21]. The prevalence rate in this study was 24.8%. The prevalence of raised LDL-c in our HAART group was 33.6%. It is similar with the prevalence reported from India [21]. We found that the prevalence of raised TG in HAART group was 55.8%. This is not in line with the prevalence reported from Cameroon [14], Kenya [15], and India [21]. The prevalence rates in these three studies were respectively, 43.5%, 22.5%, and 42.1%. However, there are suggestions that the magnitude of lipid profile derangements induced by first-line HAART could show variation with duration of treatment, across populations and setting.

The reports from rural South India and Brazil indicated that the proportion of lipid profile derangements were higher among patients received d4T when compared to other NRTIs [21,22]. We found no difference in lipid derangements when patients received d4T compared to those received ZDV. This finding is similar with the reports from Uganda, and Cameroon [11,14]. The randomized trial report of 2 non-nucleosides (2NN) indicated that patients on NVP group had significantly improved HDL-c concentration and had relatively low lipid profile derangements when compared to those on EFV [23]. In line with the reports from rural Uganda [11], Cameroon [14] and India [24], we found no significant difference in lipid profile of patients on EFV compared to those on NVP. In the present study, the raised TC and LDL-c were significantly and positively associated with the use of HAART treatment, and the findings are in line with the study conducted in Cameroon [14].

### Limitations of the study

Comprehensive cardiovascular risk stratifications were not assessed in this study. However, the increased risk of cardiovascular diseases associated with described lipid derangements is well known [25-27], and long term use of first-line HAART may have an impact on cardiovascular system. The other limitations were the cross-sectional nature of the study, small number of male participants, and lack of HIV negative controls.

## Conclusion

Our study indicates HIV-infected patients receiving WHO-recommended first-line HAART that include Efavirenz and Nevirapine have a high prevalence of lipid profile derangements when compared to those non-treated HIV-infected patients. Uses of first-line HAART regimens are significantly associated with atherogenic lipid profiles. Therefore, the findings indicate that the need to assess lipid profiles at baseline before initiation of HAART treatment and lipid profile monitoring during therapy to monitor any rising trends. Additionally, the results also recommend implementation of well-controlled cohort studies for the evaluation of long-term effects of HAART treatment on lipid profiles.

## Abbreviations

2NN: 2 non-nucleosides; AIDS: Acquired immunodeficiency syndrome; HAART: Highly active antiretroviral therapy; ZDV: Zidovudine; d4T: Stavudine; EFV: Efavirenz; ART: Antiretroviral therapy; HDL-c: HDL-cholesterol; 3TC: Lamivudine; HIV: Human immunodeficiency virus; WHO: World Health Organization; LDL-c: LDL-cholesterol; NNRTIs: Non-nucleoside reverse transcriptase inhibitors; NVP: Nevirapine; PIs: Protease inhibitors; TC: Total cholesterol; TG: Triglycerides; SPSS: Statistical package for social sciences; USNCEP-ATP: United States National Cholesterol Education Program, Adult Treatment Panel.

## Competing interests

We announce that we have no any competing interests.

## Authors’ contributions

AT generated and designed the study, performed analysis and interpretation of data including with manuscript drafting, ZA, HA and SB assisted with the design, interpretation of data and the critical appraisal of the manuscript. All authors read and agreed the final manuscript.
